# MVS-T: A Coarse-to-Fine Multi-View Stereo Network with Transformer for Low-Resolution Images 3D Reconstruction

**DOI:** 10.3390/s22197659

**Published:** 2022-10-09

**Authors:** Ruiming Jia, Xin Chen, Jiali Cui, Zhenghui Hu

**Affiliations:** 1 School of Information Science and Technology, North China University of Technology, Beijing 100144, China; 2 Hangzhou Innovation Institute, Beihang University, Hangzhou 310051, China

**Keywords:** multi-view stereo, transformer, 3D reconstruction, attention mechanism

## Abstract

A coarse-to-fine multi-view stereo network with Transformer (MVS-T) is proposed to solve the problems of sparse point clouds and low accuracy in reconstructing 3D scenes from low-resolution multi-view images. The network uses a coarse-to-fine strategy to estimate the depth of the image progressively and reconstruct the 3D point cloud. First, pyramids of image features are constructed to transfer the semantic and spatial information among features at different scales. Then, the Transformer module is employed to aggregate the image’s global context information and capture the internal correlation of the feature map. Finally, the image depth is inferred by constructing a cost volume and iterating through the various stages. For 3D reconstruction of low-resolution images, experiment results show that the 3D point cloud obtained by the network is more accurate and complete, which outperforms other advanced algorithms in terms of objective metrics and subjective visualization.

## 1. Introduction

Multi-view stereo (MVS), a significant field in computer vision, intends to reconstruct 3D models with dense representations from multi-view images and related internal and external camera parameter matrices. The research results of 3D reconstruction have been frequently used in robot navigation [1,2], augmented reality [3], and photogrammetry [4,5]. High-resolution images allow for better reconstruction, but such large-size images consume a lot of processing resources and time. However, mobile robots have high requirements for real-time 3D reconstruction. Therefore, how to effectively and rapidly reconstruct a 3D model from low-resolution images that lack detailed information but retain a complete structure is what we are considering, which will contribute to the next step of high-resolution reconstruction work.

The traditional MVS algorithms rely on hand-crafted similarity metrics [6,7], and are optimized with regularizations such as semi-global matching to generate dense point clouds. However, these methods rely on ideal Lambertian surfaces, and there are still limitations on the completeness and scalability of the reconstruction [8,9]. To address the above problem, we aim to investigate a more accurate and straightforward 3D reconstruction method for low-resolution images.

Learning-based methods have obtained impressive results in MVS tasks [10,11,12,13,14,15,16,17]. Typically, convolutional neural networks (CNN) are used in these methods to extract image features and then warped source image features to the reference camera frustum to produce cost volume, which is utilized to predict the depth map of each view. Finally, the 3D point cloud can be generated by fusing the multi-view depth map. This pipeline decouples the MVS task into a regression problem between the multi-view image and the depth map, resulting in higher reconstruction accuracy than traditional methods. The convolution-based backbone gradually downsamples the image, extracting multi-scale features and using receptive fields of various sizes to progressively abstract low-level characteristics into high-level features, capturing the image’s local attributes. However, feature resolution and granularity lost in the deeper stages of the model are not conducive to the reconstruction of low-resolution images.

Recently, neural network design in natural language processing (NLP) has embarked on a completely different path since Transformer [18] has replaced recurrent neural networks as the dominant network architecture. With the introduction of Vision Transformers (ViT) [19], more and more scholars apply Transformer to computer vision [20,21,22,23,24,25]. The Transformer’s superior design architecture and self-attention mechanism can better model spatial relationships and aggregate features at arbitrary locations.

Therefore, we propose an innovative neural network architecture with Transformer for deep inference in the MVS task. The network uses encoder-decoder architecture for low-resolution image reconstruction. The image feature pyramid is first extracted using the Three-stage Feature Aggregation module (TFA), which focuses on semantic and shallow information at the pixel level. Then, Transformer is applied to the coarsest resolution features, using self-attention to enhance the long-term global context awareness of the image. To better apply the Transformer architecture in the MVS task, we recombined the bag of words representation provided by ViT into an image-like feature representation. Finally, following the coarse-to-fine volume regularization pattern [11], the feature volume is decoded, and a dense 3D reconstruction is performed.

The key contributions of this study can be categorized into three aspects. First, a coarse-to-fine MVS network with Transformer (MVS-T) is proposed for MVS reconstruction of low-resolution images. Second, the three-stage feature aggregation module is proposed to merge multi-scale image features and preserve structural and detailed information to improve depth estimation accuracy. Then, after validating different variants of ViT, the vanilla Transformer block is introduced for global context information perception. The fusion module recombines the Transformer outputs into image-like features to capture dependent information for subsequent deep inference. Third, through detailed experiments on the MVS task dataset DTU [8], the proposed method increases the precision of low-resolution image 3D reconstruction, which is superior to other advanced algorithms.

The structure of this study is organized as follows. In Section 2, we discuss the related work on multi-view stereo reconstruction. We introduce the detail of our method in Section 3. In Section 4, we assess the performance of the proposed algorithm. In Section 5, we present our conclusions.

## 2. Related Work

### 2.1. Multi-View Stereo Reconstruction

Research related to MVS has been conducted for decades. The traditional methods mainly include Structure from Motion (SfM) [26,27] and Simultaneous Localization and Mapping (SLAM) [28]. Both SfM and SLAM can achieve good 3D reconstruction results, but they rely on feature matching, which becomes very difficult when the distance between multi-view is too large.

Deep learning-based methods are developing rapidly, driving progress in tasks including target detection [29], depth estimation [30], and image deblurring [31]. Convolutional neural networks have considerable advantages in feature matching of images and do not require complex camera calibration, so they have attracted great interest in 3D reconstruction. Learning-based methods tend to restore dense 3D surfaces from features of multi-images and perform better in 3D reconstruction.

SurfaceNet [32] is the first learning-based pipeline for MVS tasks. It uses a series of images and the associated camera parameter matrix as input, directly obtaining surface voxels as output. The literature [10] proposed an MVS method for large-scale scene reconstruction, using a 2D-CNN encoder and a 3D-CNN decoder to perform deep inference on each view of the input and then outputting a 3D point cloud model by a fusion module. CasMVSNet [14] uses a coarse-to-fine multi-stage approach to predict the corresponding coarse depth map at low resolution and then builds on this with higher resolution features to narrow down the depth hypotheses to optimize the depth map. Compared to the method of volumetric representations [33], the depth map-based MVS method dramatically improves the flexibility of 3D scene reconstruction and reduces memory consumption. Therefore, we also adopted the depth map representation for 3D reconstruction.

### 2.2. Transformer

The Transformer architecture introduced by Vaswani et al. [18] has become a reference model in NLP tasks. Inspired by this, Transformer variants for various studies have been proposed. Among them, ViT [19] applied the Transformer architecture to image classification for the first time, and with the help of large-scale datasets, its accuracy has surpassed convolutional networks. DeiT [34] introduced distillation methods into the training of ViT, used a teacher–student training strategy, and proposed a distillation token to improve the model’s performance. Swin-T [35] built a general framework for vision tasks, which can be used for target detection and semantic segmentation. These attempts have been successful in image classification and have shown promising applications of Transformer. Transformer architecture is starting to be applied in MVS. TransMVSNet [22] introduced inter- and intra-attention, focusing on both cross- and self-image information. MVSTR [23] designed a global-context Transformer and a 3D-geometry Transformer to facilitate information interaction. MVSTER [24] proposed epipolar Transformer for 3D spatial correlations and used geometric knowledge to build the correlation along epipolar line to improve model efficiency. WT-MVSNet [25] utilized epipolar constraints to reduce redundant information and enhance patch-to-patch matching. Contrarily, our MVS-T does not introduce additional constraints or elaborate complex structures, but has performed well in our task.

The Transformer model, based on the self-attention mechanism, can capture the internal correlation of features and retain positional relationships during feature propagation, facilitating the perception of global context information. These natural advantages of the Transformer enable it to complement the shortcomings of the CNN approach and allow it to fulfill its potential in the MVS task.

## 3. Methods

**Figure 1:**
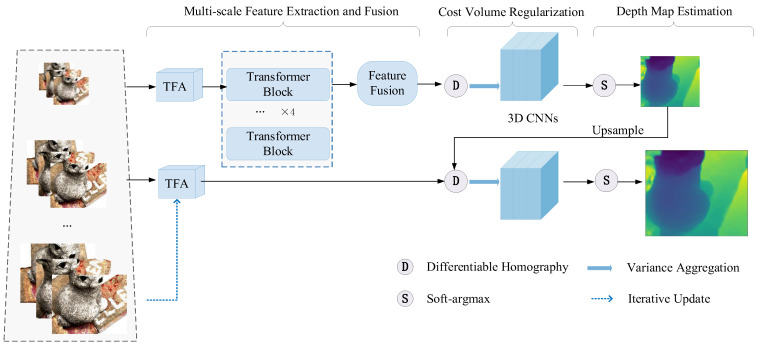
The network structure of MVS-T.

Figure 1 depicts the detailed structure of MVS-T. The network’s input consists of the reference image $I_{0}\in {\mathbb{R}}^{H\times W}$, the source images ${\left\{I_{i}\right\}}_{i=1}^{N}$, and the camera intrinsic, rotation matrix, and translation vector of the corresponding view ${\left\{K_{i},R_{i},t_{i}\right\}}_{i=0}^{N}$. The output is the depth map **D** for reference image **I**_0_. After performing a photometric consistency check and filtering on the depth maps of all views, we finally generated a 3D point cloud. The originality of our method lies in focusing on the shallow information of the images in the multi-stage process and applying the Transformer architecture in the MVS task to improve the global context perception of each view. In the following, we will describe the details of the feature pyramid, Transformer global perception module, image-like feature resampling, cost volume construction, and loss function in our approach.

### 3.1. Image Feature Pyramid

**Figure 2:**
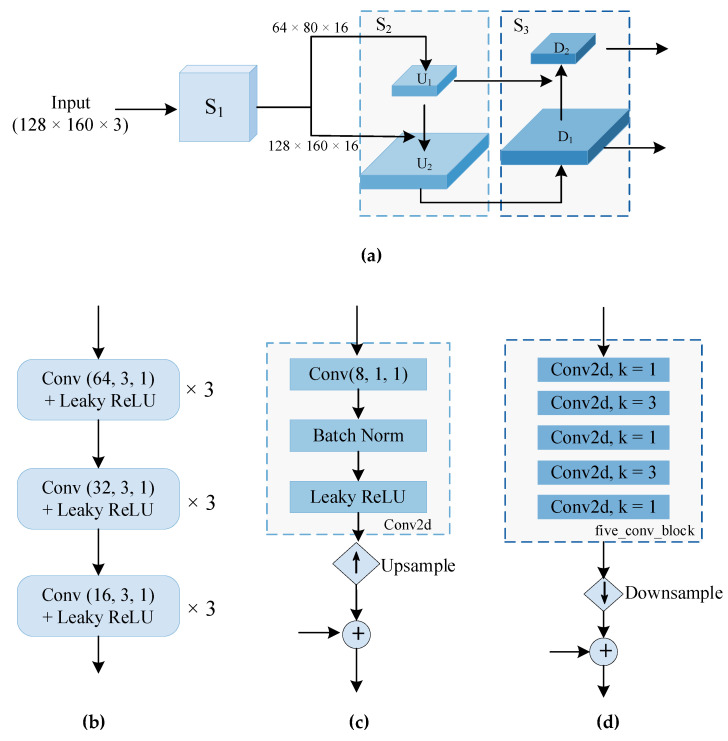
The structure of TFA module. (**a**) TFA; (**b**) S_1_; (**c**) S_2_−U; (**d**) S_3_−D.

The input raw image will be influenced by environmental factors such as illumination, and we use learnable features that are widely used in dense prediction tasks to extract abstract semantic information from the initial image. The overall process of feature extraction is shown in Figure 2a, which is divided into three stages.

Figure 2b illustrates the specific structure of the first stage. An *L*-level image pyramid ${\left\{I_{i}^{j}\right\}}_{j=0}^{L-1}$ was constructed for every input view, i∈{0,1,…,N}, which represents the input (*N*+1) images. Then, features were extracted from the input image using a neural network consisting of nine convolutional layers containing a rectified linear unit (ReLU), with a ratio of *2* for the feature mapping between stages. We define the *l*-level features as ${\left\{f_{i}^{l}\right\}}_{i=0}^{N}$, where $f_{i}^{l}\in {\mathbb{R}}^{\frac{H}{2^{l}}\times \frac{W}{2^{l}}\times F}$, *H* and *W* denote the initial input image size, and *F* refers to the number of feature channels output after stage one, which is set to 16 in this paper.

However, for low-resolution images, the pyramid structure enables top-level features to obtain high-level semantic information while ignoring information in the shallow layers, which is not conducive to the subsequent dense prediction. To this end, we use a lateral connection structure similar to U-Net [36]. In the second stage of the top-down pyramid process, the feature of the upper layer is upsampled to obtain the same size as the current layer. It is fused with the feature in the corresponding level of the first stage by using concatenation through lateral connection. The specific structure is shown in Figure 2c, where the upper layer feature $f^{l}$ passes through a 1 × 1 convolutional layer containing a batch-normalization operation and Leaky ReLU to obtain $c^{l}$. Then, the small-size feature $c^{l}$ is upsampled by nearest neighbor interpolation and concatenated $f^{l-1}$ through the 1 × 1 convolutional layer to obtain $c^{l-1}$.

To improve the utilization of the low-level information and increase its propagation efficiency, we perform bottom-up information enhancement ${\left\{c_{i}^{l}\right\}}_{l=0}^{L-1}$ in the third stage. As depicted in Figure 2d, the underlying feature $c^{l-1}$ passed through a five convolution block to obtain $p^{l-1}$, then was downsampled and concatenated with the current level features $c^{l}$. Finally, a five convolution block was used to adjust the number of channels, and $p^{l}$ was obtained. The ${\left\{p_{i}^{l}\right\}}_{l=0}^{L-1}$ is the final image feature pyramid constructed in our method.

### 3.2. Transformer for Coarse Feature Fusion

The previous learning-based MVS methods build cost volume from extracted features directly, ignoring the importance of global context information for deep inference, especially in low-resolution image scenes, where information loss is severe and detrimental to 3D reconstruction. The multi-head attention (MHA) mechanism in Transformer [18] is a global operation that can focus on and affect all input tokens. Therefore, we proposed applying Transformer in the MVS task. Considering the demanding computational complexity of self-attention in the Transformer, we only used the Transformer block at the coarsest resolution.

#### 3.2.1. Transformer Block

For the convenience of subsequent representation, we define the input feature map $p_{i}^{l}\in {\mathbb{R}}^{\frac{H}{2^{l}}\times \frac{W}{2^{l}}\times F}$ as $p_{i}\in {\mathbb{R}}^{H^{\prime }\times W^{\prime }\times F}$. The expected input form of Transformer is {N,D}, *N* is equivalent to the length of the sequence in NLP, and *D* is the dimension of each token in sequence. For the computer vision task, we need to reduce the two-dimensional feature map into a one-dimensional sequence to satisfy the Transformer’s input. The first step is to divide each feature map into *N_p_* image blocks of the same size, where $N_{p}=\frac{H^{\prime }}{p}\times \frac{W^{\prime }}{p}$, and *P* = 4 is the size of each image block set in this paper, so the size of the input is reduced from $\left\{F,H^{\prime },W^{\prime }\right\}$ to $\left\{N_{p},P^{2}\times F\right\}$. In the second step, feed these *N* image blocks into the linear projection layer. In all Transformer blocks, the constant latent vector size *D* is used, so we mapped them to *D* dimensions and added position information to these *N* patch embedding. In the third step, after experimental verification, similar to BERT [37], we added a learnable embedding $t_{0}^{l}\in {\mathbb{R}}^{D}$, and the final output is $t^{l}\in {\mathbb{R}}^{\left(N_{p}+1\right)\times D}$. The exact procedure is shown in Equation (1).

(1) $$t^{l}=\left[t_{0}^{l};t_{1}^{l};\ldots ;t_{N}^{l}\right]=\left[t_{class}^{l};x_{1}^{l}E;x_{2}^{l}E;\ldots ;x_{N_{p}}^{l}E;\right]+E_{pos,}\;\;\;\;\;\;x\in {\mathbb{R}}^{P^{2}\times F},E\in {\mathbb{R}}^{\left(p^{2}\times F\right)\times D},E_{pos}\in {\mathbb{R}}^{\left(N_{p}+1\right)\times D}$$

**Figure 3:**
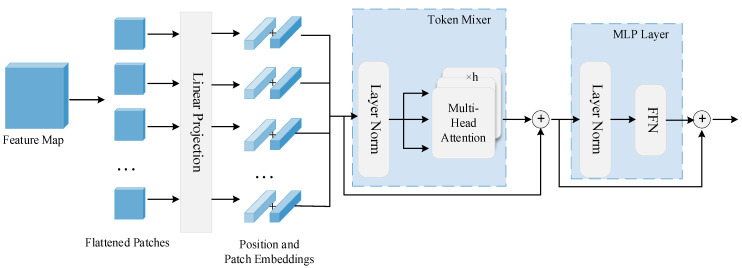
The structure of Transformer block.

The Transformer block comprises a token mixer layer and a multi-layer perceptron (MLP) layer. In Figure 3, the token mixer consists of a layer norm and the multi-head attention, while the MLP consists of a layer norm and a feedforward network containing two linear transformations. Map the input of the Transformer layer $t_{i}^{l}$ to query **Q**, key **K**, and value **V**. When the matching degree of Q and K is higher, the weight is higher. The self-attention mechanism is described in Equation (2), where *d_k_* is the dimension of **Q**, **K,** and **V**.

(2) $$SelfAtten\left(Q,K,V\right)=softmax\left(\frac{QK^{T}}{\sqrt{d_{k}}}\right)V$$

The MHA linearly projects each query, key, and value to different subspaces for *h* times with the projected dimensions *d_q_*, *d_k_*, and *d_v_*. Then, as shown in Equation (3), after performing *h* self-attention calculations, concatenate the results obtained each time.

(3) $$MultiHeadAtten(Q,K,V)=Concat(SelfAtten_{1},\ldots ,SelfAtten_{h})$$

Finally, through the MLP layer, the final output is obtained after the residual connection. In this paper, we set the number of Transformer blocks to 4.

#### 3.2.2. Image-like Feature Fusion Module

**Figure 4:**
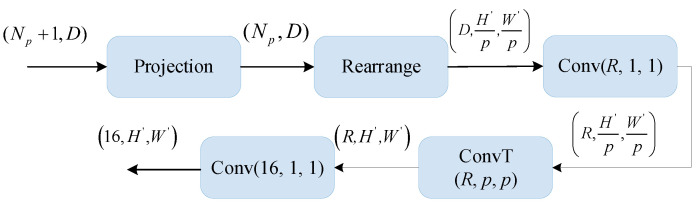
The structure of an image-like fusion module.

The Transformer block outputs a set of patch embeddings. When applied to image-dense prediction tasks, we need to re-fuse them into the representation of image-like features. Based on this, we designed an image-like feature fusion module, which is used to gradually convert the embedding output by the Transformer into image-like feature maps. The overall flow of this fusion module is shown in Figure 4.

The input of this fusion module is (*N_P_* + 1) patch embeddings, where *N_p_* patches ${\left\{t_{i}^{l}\right\}}_{i=1}^{N}$ are extracted from the initial image, and the remaining one $t_{0}^{l}$ is added manually. The $t_{0}^{l}$ is generally used for the final classification or detection in vision tasks, and we explored its effectiveness in the MVS task. The randomly initialized classification embedding encodes the characteristics of the whole dataset and avoids bias. For the input (*N_p_* + 1) embeddings, we map them to *N_p_* and then reset the tensor using rearrange operation. According to the position of the initial patches in the image, a feature map with the size of $\frac{H^{\prime }}{p}\times \frac{W^{\prime }}{p}$ is obtained. The dimension of channels is adjusted to *R* by using 1 × 1 convolution, and the scale is restored using a transposed convolution with both the kernel size and step size of 4 to return to the original input feature shape $H^{\prime }\times W^{\prime }$. The input embeddings have been converted to a feature map with a specific size, which can be used for subsequent image tasks.

### 3.3. Depth Inference for MVS

Referring to previous approaches [10,11,12], we used the plane scanning principle to generate the cost volumes and infer the depth of the reference view from the input (*N* + 1) feature maps. Because the construction of the 3D cost volume and the computation of self-attention in the Transformer block consume a large amount of memory, we adopt a multi-stage approach from coarse-to-fine, build the cost volume pyramid, and gradually refine the depth map estimation.

Similar to MVSNet [10], ${\left\{K_{i},R_{i},t_{i}\right\}}_{i=0}^{N}$ is the camera intrinsic, rotation matrix, and translation vector for the corresponding feature map. When *i* = 0, it is denoted as ref view, and the rest is source view. For different stages, we used the differentiable homography to warp the source image’s feature map to the reference view after setting *M* depth hypotheses *d*. The differentiable homography is calculated as:

(4) $$H_{i}^{l}\left(d\right)=K_{i}^{l}R^{i}\left(I-\frac{\left(t_{0}-t_{i}\right)n_{0}^{T}}{d}\right)R_{0}^{-1}{\left(K_{0}^{l}\right)}^{-1},$$

where the scaled camera intrinsic of feature map corresponding to the *l* level pyramid denote as $K^{l}$, and **I** being the unit matrix. Given the camera parameters and the depth hypotheses *d*, the possible correspondence of pixels between the different views can be found.

A source image is warped to different depths to form a feature volume. A cost volume is constructed by aggregating the variance of the *N* source image feature volumes and the reference feature volume. After regularizing the cost volume, a probability volume is produced using the 3D convolutional decoding network [11]. The depth of each pixel can be calculated from Equation (5) by multiplying the probability of the pixel at the corresponding depth with that depth and then summing the results at different depths to get the final pixel-level depth value. The depth hypotheses are further narrowed using the coarsest resolution depth map as an a priori, and the depth map is constantly refined by building a cost volume pyramid.

(5) $$D^{l}\left(p\right)=\sum_{m=0}^{M-1}dP_{p}^{l}(d)$$

### 3.4. Loss Function

Like other coarse-to-fine multi-stage MVS methods, we sampled the ground truth depth into the corresponding level pyramid and employ *L*1 loss as the supervision signal to compute the absolute distance between the ground truth depth and the predicted depth. The loss function is defined as follows:

(6) $$L=\sum_{l=0}^{L-1}\sum_{p\in \Omega }{\left\|D_{GT}^{l}\left(p\right)-D^{l}(p)\right\|}_{1}$$

where Ω is the set of valid pixels, *GT* is the ground truth, and *l* denotes the *l*-th level of the pyramid.

## 4. Experiments

### 4.1. Dataset

We used the publicly available DTU dataset [8] to train and evaluate our model. The dataset utilizes an industrial robot arm mounted with a structured light scanner to capture multiple views of an object and provides a reference 3D surface geometry of the viewed object. The camera position is strictly controlled, and the camera parameters of each view can be obtained.

The DTU dataset contains 124 scenes from 49 or 64 positions under 7 lighting conditions, from directional to diffuse. To verify the effectiveness of the proposed algorithm, we followed the previous methods [10,11] to divide the training set and the evaluation set. The training set consisted of 79 scenes, and the evaluation set contained 22 scenes, each recording 49 images from different angles.

BlendedMVS dataset [38] is a novel large-scale synthetic dataset, containing more than 17k MVS training samples and 113 scenes. However, this dataset does not provide ground truth point clouds, and there is no pipeline for point cloud evaluation. Therefore, we only used the BlendedMVS dataset to qualitatively display the visualization results.

### 4.2. Metrics

In the MVS task, some commonly used metrics evaluate the difference between the reconstructed point clouds and the ground truth point clouds. We chose *accuracy*, *completeness*, and *overall score* to evaluate our algorithm. *Accuracy* calculates the distance between the predicted 3D points and the true value provided by the structured light sensor in millimeters. *Completeness* reports the distance between the ground truth value and the predicted points, which measures the integrity of the MVS reconstruction [39]. Since *accuracy* and *completeness* are a pair of trade-off metrics, to avoid the situation where only high-precision points are retained to improve the accuracy of the algorithm while ignoring the integrity of the reconstructed scene, we used the *overall* to calculate the average score of *accuracy* and *completeness*. In MVS 3D reconstruction, these lower metrics indicate higher model performance.

### 4.3. Implementation Details

We implemented MVS-T with PyTorch and trained it on an NVIDIA GeForce TITAN RTX GPU with 24 GB memory. We used Adam [40] to optimize the proposed method with hyperparameters $\beta_{1}=0.9$, $\beta_{2}=0.999$. We set the batch size to 16 and trained 27 epochs. The initial learning rate is set to 0.001 and decayed by a factor of 0.5 after the 10th, 12th, 14th, and 20th epochs.

In training, we adopted three views with the resolution of 160 × 128 as inputs to build a two-level pyramid. For the coarsest resolution level, the *M* = 48 depth hypotheses were uniformly sampled from 425 mm to 935 mm. In the next level, we set *M* = 8 for depth refinement, since the coarse depth map predicted at the previous level provides a priori. According to the literature [10], we used [41] to fuse the depth maps, generate a dense point cloud, and then used the MATLAB script provided by the DTU dataset for metric evaluation.

### 4.4. Experimental Performance

#### 4.4.1. Results on DTU Dataset

In the evaluation phase, we set the input views to 3 and the image size 160 × 128. This section compares the method we proposed with other learning-based MVS approaches. The comparison results on objective metrics are shown in Table 1.

Colmap [7] is a traditional MVS pipeline which can incrementally reconstruct 3D models by finding the corresponding relationship between image pairs. However, the matching points of this method are sparse in low-resolution images. AA-RMVSNet [13] presents an adaptive aggregation recurrent MVS network that uses long short-term memory (LSTM) and performs with better accuracy. In addition to current stereo matching algorithms based on 3D cost volumes, CasMVSNet [14] presents a cascade approach to save memory and time. CVP-MVSNet [11] infers high-resolution depth maps using a compact, lightweight network for better reconstruction performance. AACVP-MVSNet [12] introduces the attention layer to improve feature extraction ability and uses similarity metrics to aggregate cost volumes, which performs best in completeness. MVSTER [24] and TransMVS [22] are both Transformer-based methods. Compared to other advanced methods, our algorithm trades off accuracy and completeness and achieves the best result in the *overall* metric.

**Figure 5:**
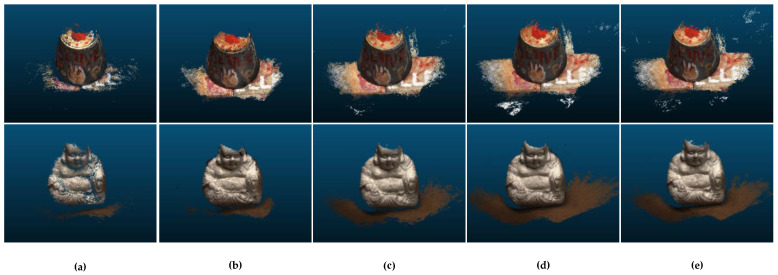
Comparison of reconstructed results. (**a**) AA-RMVSNet; (**b**) CasMVSNet; (**c**) CVPMVSNet; (**d**) AACVP-MVSNet; (**e**) Ours.

The visual comparison is shown in Figure 5. The reconstruction results of AA-RMVSNet are demonstrated in Figure 5a, which retains relatively accurate points in exchange for accuracy at the cost of integrity, resulting in a sparse reconstructed point cloud. Figure 5b,c represent the reconstruction results of CasMVSNet and CVPMVSNet, respectively, both of which use a coarse-to-fine approach to increase the reconstruction quality while reducing memory consumption. The reconstructed point cloud of AACVP-MVSNet in Figure 5d is more complete but compared with our results in Figure 5e, the noise is more, and accuracy is lower. Thus, the 3D reconstruction of low-resolution images using our method produces good visualization results.

#### 4.4.2. Results on BlendedMVS Dataset

**Figure 6:**
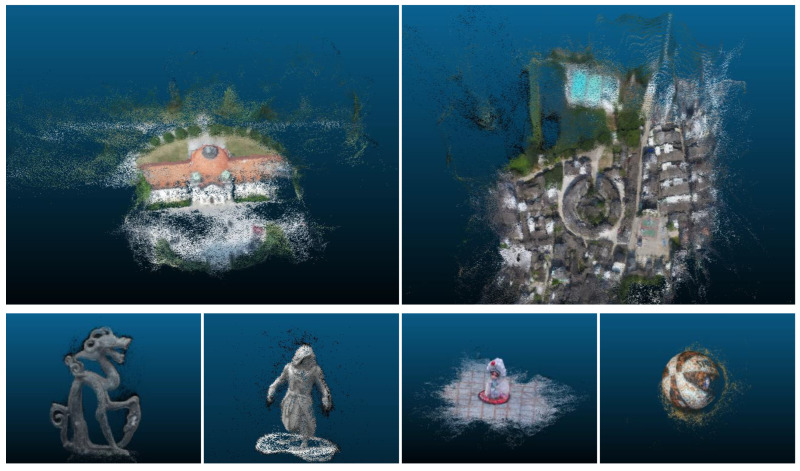
Reconstruction results of BlendedMVS dataset.

To evaluate the generalization of the proposed MVS-T, we used the model trained on DTU dataset without any fine-tuning to reconstruct 3D scenes in the BlendedMVS dataset. The input images were resized to 160 × 128 and the camera parameters were scaled correspondingly. Figure 6 shows the 3D reconstruction results of our method on the BlendedMVS. The top row shows the results of the outdoor large scene, and the bottom row is the sculpture and small objects. Although the input is low-resolution images, the scene complexity span is large, and the shooting trajectories are different, our method can still complete the 3D points reconstruction of different scenes.

### 4.5. Ablation Study

#### 4.5.1. Effectiveness of Different Components

We used the TFA module to build the feature pyramid, focusing on high-level and low-level image information, and the Transformer blocks to make the networks pay more attention to global image information, effectively improving the accuracy of the reconstructed scene. We conducted ablation experiments to evaluate the effectiveness of the modules suggested in this paper, and the results are displayed in Table 2. Compared with the initial model, the complete model we proposed is 22.3% lower in *accuracy* and 4.25% lower in *completeness*.

#### 4.5.2. Evaluation Patch Size Settings

The Transformer needs to divide the input into fixed patches, and we studied the influence of patch sizes in Table 3. It can be seen that when the size of the patch is too large or too small, the performance will decrease. Therefore, patch size = 4 achieves the optimum in all objective metrics.

**Figure 7:**
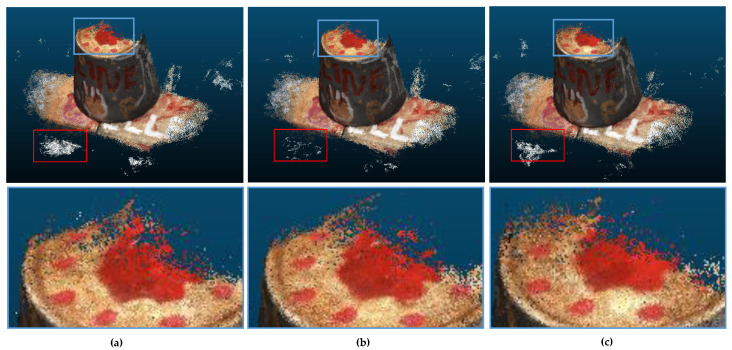
Comparison of reconstructed results. (**a**) Patch size = 2; (**b**) Patch size = 4; (**c**) Patch size = 8.

We visualized and compared their reconstructed 3D point cloud for different patch sizes, and the results are shown in Figure 7. The red box indicates that the 3D point cloud has less noise and higher accuracy when the patch size = 4. From the images in the blue box, we can see the completeness of the reconstructed point cloud under different patch sizes. When the patch size = 2, the point cloud is sparser, and when the patch size = 8, the point cloud integrity is low.

#### 4.5.3. Explore on Learnable Token

As mentioned in Section 3.2.2., we explored the validity of adding a classification token similar to BERT [37] and different fusion methods from token embeddings to image-like features. The results are shown in Table 4. The -*cls* means no additional classification token is added. The *ignore* means a classification token is added for training but directly ignored during feature fusion, the *add* means adding the classification token to other tokens, and the *map* concatenates this classification token with the rest of the tokens. As seen in the table, the classification token can guide the model to better focus on the information of the whole dataset and improve the metrics.

#### 4.5.4. Number of Different Transformer Blocks

To select the appropriate number of Transformer blocks, we adjusted the Transformer blocks *T* and conducted experiments. As is demonstrated in Table 5, *T* = 4 achieves best in all indicators.

#### 4.5.5. Extension on Different Resolution Images

We applied our proposed model on different resolution images, and the results are shown in Table 6. However, through the experiments, we find that the accuracy of the reconstructed point cloud is improved, but the improvement in completeness is not significant. This may be due to the fact that we only use low-resolution images during training, and there is insufficient extraction of high-resolution image details.

## 5. Conclusions

To reconstruct high-quality 3D scenes from low-resolution multi-view images, we propose a Transformer based multi-stage MVS network (MVS-T). The method focuses on shallow information while building pyramid features and applies Transformer self-attention to perceive global context features, providing more practical information for 3D reconstruction. Experimental results have shown that our method outperforms other advanced works on low-resolution image 3D reconstruction, balancing the accuracy and completeness of the reconstructed point clouds. Although our method achieves good results in the MVS reconstruction of low-resolution images, limited by the computational overhead, we did not discuss 3D reconstruction at high-resolution. In the future, we will attempt to design a lightweight and compact network to explore MVS tasks on high-resolution images.

## Figures and Tables

**Table 1:** Comparison of reconstruction quality in objective metrics.

Method	Acc. (mm)	Comp. (mm)	Overall (mm)
Colmap [7]	6.5778	10.1405	8.2930
AA-RMVSNet [13]	0.8207	3.4115	2.1161
CasMVSNet [14]	1.4045	1.6096	1.5071
CVP-MVSNet [11]	1.1964	1.0569	1.1267
AACVP-MVSNet [12]	1.1329	0.8814	1.0071
MVSTER [24]	2.6132	1.9704	2.2918
TransMVS [22]	1.0248	1.3075	1.1662
Ours	0.9296	1.0120	0.9708

**Table 2:** Quantitative performance with different components.

Model Settings			Mean Distance		
	TFA	Transformer	Acc.	Comp.	Overall
(a)			1.1964	1.0569	1.1267
(b)	√		0.9635	1.0257	0.9946
(c)	√	√	0.9296	1.0120	0.9708

**Table 3:** Ablation study on the size of patch on DTU dataset.

	Acc.	Comp.	Overall
patch size = 8	1.0182	1.1022	1.0602
patch size = 4	0.9296	1.0120	0.9708
patch size = 2	0.9465	1.0237	0.9851

**Table 4:** Ablation study on the learnable token and image-like fusion methods.

	*-cls*	*ignore*	*add*	*map*
Acc.	0.9287	0.9296	0.9856	0.9724
Comp.	1.0363	1.0120	1.0692	1.0505
Overall	0.9825	0.9708	1.0274	1.0114

**Table 5:** Ablation study on the number of Transformer blocks.

T	Acc.	Comp.	Overall
6	0.9731	1.0575	1.0153
4	0.9296	1.0120	0.9708
2	1.0148	1.0824	1.0486

**Table 6:** Results on different resolution images.

Image Size	Acc.	Comp.	Overall
160 × 128	0.9296	1.0120	0.9708
320 × 256	0.7695	1.0163	0.8929
640 × 512	0.5348	1.2394	0.8871
1280 × 1024	0.4089	0.9584	0.6836

## Data Availability

Not applicable.
